# Life on Minerals: Binding Behaviors of Oligonucleotides on Zirconium Silicate and Its Inhibitory Activity for the Self-Cleavage of Hammerhead Ribozyme

**DOI:** 10.3390/life12111689

**Published:** 2022-10-24

**Authors:** Kunio Kawamura, Jean-François Lambert, Louis M. P. Ter-Ovanessian, Jacques Vergne, Guy Hervé, Marie-Christine Maurel

**Affiliations:** 1 Department of Human Environmental Studies, Hiroshima Shudo University, 1-1-1 Ozuka-higashi, Asaminami-ku, Hiroshima 731-3195, Japan; 2 Laboratoire de Réactivité de Surface—CNRS UMR 7197, Campus Pierre et Marie Curie, Sorbonne Université, 7 Quai Saint-Bernard, 75005 Paris, France; 3 Institut de Systématique, Evolution, Biodiversité (ISYEB), UMR 7205 CNRS MNHN UPMC EPHE, Muséum National d’Histoire Naturelle, Sorbonne Universités, CP. 50, 57 rue Cuvier, 75005 Paris, France; 4 Laboratoire BIOSIPE, Institut de Biologie Paris-Seine, Sorbonne Université, 7 quai Saint-Bernard, 75005 Paris, France

**Keywords:** hammerhead ribozyme, chemical evolution, mineral, adsorption, Hadean Earth, zirconium silicate, montmorillonite, Aerosil, sepiolite

## Abstract

The role of minerals in the chemical evolution of RNA molecules is an important issue when considering the early stage of the Hadean Earth. In particular, the interaction between functional ribozymes and ancient minerals under simulated primitive conditions is a recent research focus. We are currently attempting to design a primitive RNA metabolic network which would function with minerals, and believe that the simulated chemical network of RNA molecules would be useful for evaluation of the chemical evolution from a simple RNA mixture to an RNA-based life-like system. First, we measured the binding interactions of oligonucleotides with four types of minerals; Aerosil silica, zirconium silicate, sepiolite, and montmorillonite. Oligonucleotides bound zirconium silicate and montmorillonite in the presence of MgCl_2_, and bound sepiolite both in the presence and absence of MgCl_2_, but they did not bind Aerosil. Based on the binding behavior, we attempted the self-cleavage reaction of the hammerhead ribozyme from an avocado viroid. This reaction was strongly inhibited by zirconium silicate, a compound regarded as mineral evidence for the existence of water. The present study suggests that the chemical evolution of functional RNA molecules requires specific conformational binding, resulting in efficient ribozyme function as well as zirconium silicate for the chemical evolution of biomolecules.

## 1. Introduction

The RNA world hypothesis is considered to explain how simple chemical networks of prebiotic molecules became something that could be regarded as a life-like system [1,2]. For the evolution of such systems, natural environments with different temperatures and pressures, and with different minerals, should be considered for the chemical evolution of RNA molecules. Several successful studies, including prebiotic chemistry experiments on the formation of RNA molecules [3,4] and in vitro selection of functional RNA molecules [5,6] support the presence of an RNA-based life-like system on the primitive Earth. Therefore, realistic simulations on Hadean environments at different temperatures and pressures in the presence of minerals are important for evaluation of the steps leading from simple RNA monomers to functional RNA molecules.

We, and other groups, have developed different types of research tools that enable measurements of the behavior of biomolecules at high temperature and pressure [7,8], and information regarding the chemical and biochemical reaction behaviors of nucleotides, oligonucleotides, and more functional RNA molecules are being accumulated. We recently focused on viroids and ribozymes with respect to the chemical evolution of functional RNA [9]. Viroids are parasitic bodies that are sometimes considered as living fossils in relation to the RNA world. Recently, we showed a compensation effect of relatively high temperature and very high pressure, i.e., conditions that may be considered a realistic simulation of the deep ocean environment, for the self-cleavage of viroids and ribozymes [9,10]. This suggests that the submarine hydrothermal vent system in the deep ocean at relatively high temperature and high pressure would have been suitable for the chemical evolution of functional RNA molecules.

It is also reasonable to think that minerals would have played important roles for the accumulation and chemical evolution of RNA and related molecules. Thus, different groups have evaluated the roles of minerals for the chemical evolution of biomolecules in the presence of minerals under simulated Hadean Earth environments. Several groups have demonstrated the role of minerals for the chemical evolution of RNA and related biomolecules [11,12,13,14,15,16,17,18,19,20]. The minerals would have had concentrated organic molecules on their surface where the prebiotic reaction could have been effectively enhanced. Successful studies include examples of enhancement of the adsorption and concentration of biomolecules [21], acceleration of abiotic formation or, conversely, of the degradation of biopolymers, as well as the selection of RNA molecules [22]. In summary, minerals could have acted as pre- or proto-enzymes for the accumulation and transformation of biomolecules prior to the era when RNA molecules had taken over the role of mineral catalysts [2,23,24,25].

**Figure 1:**
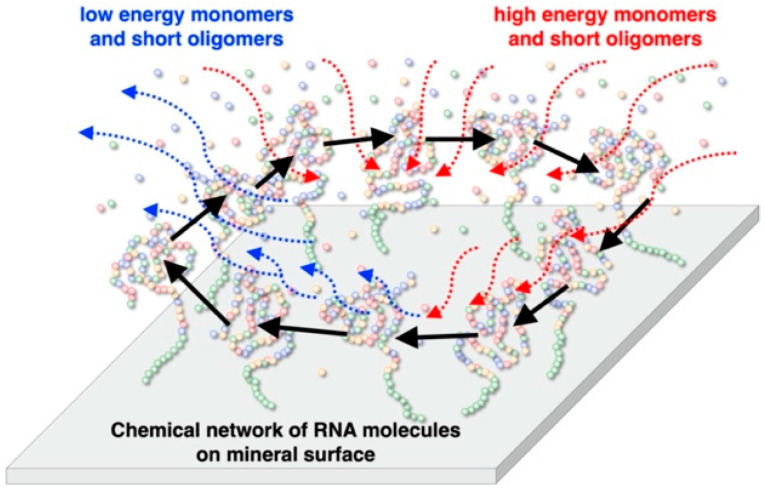
Schematic model for a primitive RNA metabolic system on a mineral surface. Short RNA molecules do not bind onto minerals strongly, while functional long RNA molecules bind onto minerals strongly. The functional RNA molecules could form a metabolic chemical network on the mineral. This can be regarded as an integrated RNA system prior to a cell-type compartmentation of RNA molecules.

Due to the importance of RNA, minerals, and hydrothermal environments, we focused on the reaction behavior of ribozyme on minerals. We are currently attempting to design a chemical network of RNA molecules on minerals under high temperature and pressure, using several functional RNA molecules adsorbed on the surface, and to compose a metabolism-like chemical network on the mineral (Figure 1). Here, we first examined the binding behavior of model oligonucleotides, which were designed to investigate whether or not the oligonucleotides adsorb effectively on four different types of minerals. Second, on the basis of the binding behaviors, we found that zirconium silicate and montmorillonite are suitable adsorbents to investigate the self-cleavage reaction of a ribozyme ASBVd(−):HHR originating from the Avocado Sunblotch viroid (ASBVd(−)) in the presence of these minerals. As a conclusion, we showed that both zirconium silicate and montmorillonite inhibit the self-cleavage reaction of ASBVd(−):HHR. Based on the results, we discuss the role of minerals for the formation of an RNA world on the Hadean Earth.

## 2. Materials and Methods

### 2.1. Materials

RNA and DNA used in the study, as shown in Table 1 and Table 2, were purchased from RiboPro., Netherland and Integrated DNA Technologies (IDT), Germany. Reagents used for ribozyme reactions were of DNase and RNase-free grade in pure water. MgCl_2_ solution, and EDTA solution were purchased from SIGMA-Aldrich, and HEPES solution was purchased from Jean Bioscience, Germany. Reagents used for HPLC analysis were of analytical grade. Aerosil^®^380 (SiO_2_) was purchased from EVONIK, Germany. Zirconium silicate (ZrSiO_4_) was purchased from CERADEL, France. A more detailed characterization of CERADEL is available in the supplementary data in CERADEL. Sepiolite (Mg_2_H_2_Si_3_O_9_·xH_2_O) and Montmorillonite K10 (pH = 3.0–4.0) were purchased from MERCK, Germany. The surface area of minerals was measured by Belsorp max, Microtrac MRB, Japan, US, Germany. Oligoadenylic acid (oligo(A)) was prepared by partial hydrolysis of polyadenylic acids (SIGMA, P-9403) in 0.1 M NaOH solution at 37 °C for 45 min and treated in 0.1 M HCl at 37 °C for 1 h to hydrolyze the 2′,3′-cyclic phosphate group at the terminal to 2′- or 3′- phosphate group. DNA molecules used for binding behavior experiments were purchased from IDT, Germany.

### 2.2. Nucleic Acid Solutions Preparation

A 400–2000 µL solution containing 1 µM DNA, 0.05 M MgCl_2_, and 0.05 M HEPES, at pH 7.5 was prepared. Then, the mixture was added to 2–20 mg of mineral. The mixture was vortexed and allowed to stand for 1–24 h. In the case of oligo(A), its binding was measured using a solution containing 48 µM oligo(A), 0.05 M MgCl_2_, and 0.05 M HEPES, at pH 7.5. The ratio of the mineral to the solution was adjusted to 20 mg/2000 µL unless otherwise noted. The mixture was vortexed and allowed to stand for 1 h or 24 h. The isotherm curve of oligo(A) binding to zirconium silicate was taken using 9.25–231 µM oligo(A), 0.05 M MgCl_2_, and 0.05 M HEPES, at pH 7.5. The mixture was vortexed and allowed to stand for 1 h or 24 h. The binding of ribozymes was carried out using a solution containing 20 µg/2000 µL, 0.05 M HEPES, 0.05 M MgCl_2_ at pH 7.5. The mixture was vortexed and allowed to stand for 2 h or 18 h. After the equilibration time, the mixture was centrifuged, and the supernatant was removed. Then, the precipitate was washed with 1600 µL of 0.0625 M EDTA solution for 1 h, the mixture was centrifuged, and the supernatant was removed. DNA and RNA used in the study were denatured and renatured to form 2-dimensional structures prior to use for binding experiment using a personal Mastercycler (Eppendorf, USA or Techne FPR0G05D, UK) at 94 °C for 1 min followed by cooling to 22 °C at a constant temperature decrease of 3 °C/min.

The absorption spectra of the supernatants from the binding experiments with DNA, oligo(A), and ribozymes were measured at 190–400 nm using a UV-vis spectrophotometer (Libra S60, Biochrom Ltd., Cambridge, UK) with a quartz cell at light path 1.0 cm. The pH of solutions was measured after the binding using pH test paper since the solution volume is very small.

### 2.3. Self-Cleavage Behaviors of ASBVd(−):HHR on Minerals

A 15 µL (3 µg) ribozyme solution containing 10 µg ribozyme in 50 µL H_2_O and 2.5 µL of 1.0 M HEPES solution was added to 0.2 mL PCR tubing. The tubing was used for thermal cycler denaturation at 94 °C and cooling to 22 °C at a constant rate (3 °C/min) to form the ribozyme secondary structure prior to the ribozyme cleavage reaction. To the PCR tubing, 2.5 µL of 1.0 M MgCl_2_ solution and 30.0 µL H_2_O were added for use with the thermal cycler adjusted to specific temperatures to run the cleavage reaction (total volume of 50 µL). The mixture was added to 0.5–10 mg of mineral. To stop the self-cleavage reaction, 10 µL of 0.5 M EDTA solution at pH 8.0 and 110 µL H_2_O was added. The mixture was allowed to stand for 1 h to wash out the ribozyme and products. This procedure was repeated two times and all the supernatants were analyzed by HPLC. Samples were kept in a freezer at below −20 °C before sample analysis.

### 2.4. HPLC Analysis for the Products of ASBVd(−):HHR Self-Cleavage

ASBVd(−):HHR (79 nucleotide units in length) was cleaved into two components of 51nt and 28nt to optimize the HPLC method for the separation of the ribozyme (79nt) and its two cleavage components (28nt and 51nt) [10,26]. HPLC analysis was carried out using an Infinity II chromatograph (Agilent, Santa Clara, CA, USA) with an anion-exchange column (diameter 4.6 mm and length 75 mm, DNA-NPR, TOSOH, Japan) at flow rate of 0.75 mL/min using a gradient of 0.375–0.975 M NaCl at pH 9.0 with 7.5 M urea and 0.02 M Tris buffer. A reaction sample aliquot of 80 µL was injected for HPLC analysis.

### 2.5. Secondary Structure Modeling of RNA and DNA

To estimate the secondary structure of model DNA and modified ribozymes, Web Servers for RNA Secondary Structure Prediction were used [27,28]. According to this modeling, the sequences of model DNA and RNA based on ASBVd(−):HHR were selected for the binding experiments. The parameters were used as default values except for changing temperature.

## 3. Results and Discussion

### 3.1. Concept of RNA Chemical Network Model on Mineral

Our concept for designing a life-like system on the mineral surface is illustrated in Figure 1. According to our previous study [29,30], it was assumed that an integrated system consisting of functional RNA molecules on a mineral surface, or inside pores, possesses essential characteristics to construct a life-like system. RNA is a candidate molecule that codes for information and has enzymatic activities. Confinement of molecules in cell-type compartments is very important in modern organisms. Presumably, the emergence of such cell-type compartments required the establishment of the formation of fatty acids and membrane proteins by chemical evolution. We assume that minerals would have played roles for integration of prebiotic molecules on their surfaces prior to the establishment of a cell-type compartment [31]. On the mineral surface, functional and long RNA molecules may be strongly adsorbed, compared to monomeric and short RNA molecules [22]. In such systems, long and functional RNA molecules stay adsorbed on the mineral surface, while high energy reactants are supplied from the aqueous phase and low energy wastes are released to the aqueous phase. The mineral surface adsorbing different functional RNA molecules may be considered to support proto-metabolic chemical activity. On the mineral surface, different functional RNA molecules construct a proto metabolic chemical network so that a possible integrated life-like system on the mineral surface can be established. RNA molecules could evolve to derive enzymatic and informational reactions on the surface, prior to the existence of cell-like compartments. According to this model, we are currently attempting to construct a chemical network using actual RNA molecules and mineral surfaces. As preparation, we investigated the adsorption behaviors of different oligonucleotides onto minerals, and attempted to run the self-cleavage reaction of a hammerhead ribozyme in the presence of minerals.

### 3.2. Selection of Oligonucleotides and Minerals

We investigated the self-cleavage reactions of a hammerhead ribozyme ASBVd(−):HHR and viroid ASBVd(−) at high pressure and temperatures [9,10,26]. So far, there have been few studies on the chemical behavior and the self-cleavage reactions of such a ribozyme and viroid on binding with minerals. Thus, we evaluated the binding behaviors of DNA and RNA using four different minerals to determine suitable conditions for running the self-cleavage reactions of ASBVd(−):HHR. In our previous studies, we observed that the adsorption of oligonucleotides on montmorillonite is dependent on the type of nucleobases and the polymer length [32,33]. The binding is determined mainly by the hydrophobicity of the nucleobases and the electrostatic interaction between phosphate groups and the surface charges. Mg^2+^ ions enhance the binding of oligonucleotides on clay surfaces by creating Mg^2+^ bridges between negative charges of phosphate groups and negative charges on the clay surface. There is a trend that longer and purine-rich oligonucleotides adsorb more strongly compared to shorter and pyrimidine-rich oligonucleotides. Based on this observation, we assumed that the addition of a –(GA)_n_– sequence to functional oligonucleotides at the 3′- or 5′-terminal would be effective in designing a supported life-like system, since the –(GA)_n_– sequences adsorb effectively on mineral surfaces.

**Figure 2:**
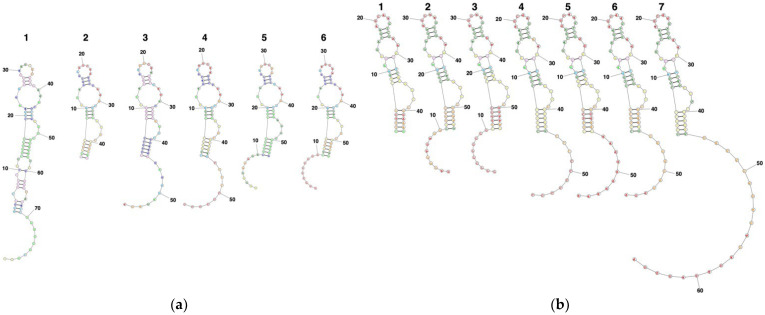
Secondary structures estimated using RNAfold WebServer for some oligonucleotides. (**a**) RNA molecules: 1: ASBVd79(−):HHR, 2: rna-ASBVd45, 3: rna-ASBVd45-^3′^(GA)_5_, 4: rna-ASBVd45-^3′^C_10_, 5: rna-ASBVd45-^5′^(GA)_5_, 6: rna-ASBVd45-^5′^C_10_; ((**b**) right) DNA molecules: 1: dna-ASBVd45, 2: dna-ASBVd45-^5′^(GA)_5_, 3: dna-ASBVd45-^5′^C_10_, 4: dna-ASBVd45-^3′^C_10_, 5: dna-ASBVd45-^5′^A_10_, 6: dna-ASBVd45-^3′^(GA)_5_, 7: dna-ASBVd45-^3′^(GA)_10_. The calculations were carried out at 328.15 K for RNA and 298.15 K for DNA. Most parameters were applied as default values, such as maximum loop size at 30, maximum energy difference at 10%, maximum number of structures at 20, and minimum helix length at 3. The predicted lowest free energy structures are displayed on the basis of the lowest free energy structure since the structures are basically the same as the structure of composed of highly probable base pairs.

We examined possible sequences of DNA and RNA suitable to form similar secondary structures, such as ASBVd(−):HHR. It is known that a core sequence is preserved in the hammerhead ribozyme [34], such as ASBVd(−):HHR and CChMVd(+):HHR [26,35].The estimation of secondary structures for RNA was carried out at 55 °C, since this temperature was the optimum for the ASBVd(−):HHR self-cleavage; the results are shown in Figure 2a,b. The estimation for DNA was carried out at 25 °C, since the formation of secondary structure is weak at 55 °C. According to the secondary structure estimation, it was predicted that the oligonucleotides tested in the present study would possess the same core structures as that of ASBVd(−):HHR at 55 °C. In contrast, the addition of –G_10_– showed a totally different secondary structure from that for ASBVd(−):HHR. The secondary structure estimation using Web Servers for RNA Secondary Structure Prediction showed that the dna-ABSVd45, rna-ASBVd45, and the analogues form the same core structure as displayed in ASBVd(−):HHR. We concluded that DNA is relevant to our approach since it possesses similar chemical structures to RNA.

The four minerals selected were aerosil silica, zirconium silicate, sepiolite, and montmorillonite, each of which are of prebiotic interest and/or possess unique characteristics. Aerosil silica is very light and forms a strongly retained “adsorbed solution” phase between the aqueous phase and the solid phase [36,37]. Zirconium silicate is used as evidence for the presence of water [38,39], but the role of zirconium silicate for the chemical evolution of biomolecules has not been much studied. Sepiolite is a clay mineral possessing a unique fibrous structure, and also has been less investigated from the viewpoint of the chemical evolution of RNA. Montmorillonite is a swelling clay that has often been used in prebiotic chemistry studies and is well known as an efficient catalyst for oligomerization of oligonucleotides and oligopeptides [11,12,32]. Montmorillonite is also assumed to be important at the early stage of the Earth [40]. It should be noted that we used the acid-activated material “montmorillonite K10”, which has rather strong acidic properties.

### 3.3. Binding Behaviors of DNA

**Figure 3:**
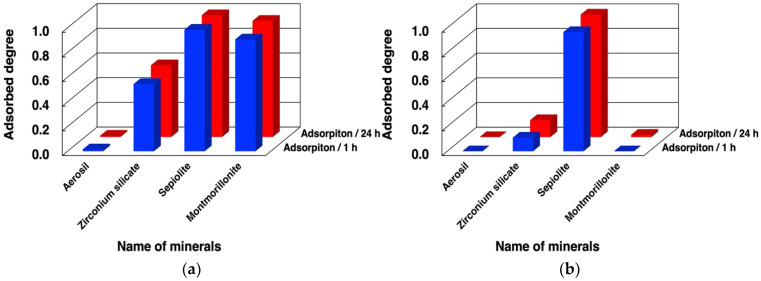
Adsorbed amounts of dna-ASBVd45 on minerals in the presence and absence of MgCl_2_. Mineral phase/aqueous phase: 20.0 mg/2000 µL, [dna-ASBVd45] = 1.14 µM, [HEPES] = 0.05 M, pH: 7.5, [MgCl_2_]: (**a**) 0.05 M, (**b**) 0 M. Adsorption time: 1 h (blue bars), 24 h (red bars).

We first examined binding of dna-ABSVd45 and its analogues to minerals under different conditions. The adsorbed fractions of dna-ASBVd45 after 1 h and 24 h on the 4 minerals are summarized in Figure 3 and in Table S1 of the supplementary data. ASBVd45 binds with montmorillonite in large amounts in the presence of MgCl_2_ and does not bind in the absence of MgCl_2_. According to our previous studies, chloride was selected as a counter ion for the magnesium ion [10,26]. In addition, dna-ASBVd45 binds onto zirconium silicate in the presence of MgCl_2_, but the binding significantly decreases in the absence of MgCl_2_. The different binding at 1 h and 24 h indicates that the binding onto these minerals is rapid. The surface areas are summarized in Table 3. The surface area of Aerosil silica, sepiolite, and montmorillonite were comparable (in the case of montmorillonite, acid activation causes a surface area increase of about an order of magnitude), but that of zirconium silicate was only a few m^2^/g. Binding of DNA onto montmorillonite in the presence and absence of MgCl_2_ was consistent with our previous studies where the Mg^2+^ ion formed bridges between phosphate groups and the negative charges of montmorillonite for binding of oligonucleotides. The fact that the binding onto zirconium silicate was also affected by Mg^2+^ suggests that the binding mechanism could be similar to that on montmorillonite even though their surface areas were very different, and thus the surface density of adsorbed DNA would be higher on the former (see § 3.4 below). Zirconium silicate is a tectosilicate, rather than a phyllosilicate like montmorillonite, but it is likely that deprotonation of its surface silanols could create a negative surface charge that would act in a similar way to that of montmorillonite [32,41,42,43]. Based on the literature [41], the surface charge was assumed to be negative at pH 7.5 since Cu^2+^, Cd^2+^, and Pb^2+^ adsorb on zirconium silicate at pH 7.0. This supports that our binding model of oligonucleotides bridged with Mg^2+^ can be applied to zirconium silicate. However, in contrast to montmorillonite, the adsorption of dna-ASBVd45 was not completely suppressed in the absence of MgCl_2_. The adsorption mechanism on this surface must be complex and should be the object of further investigation.

ASBVd45 did not bind at all onto aerosil silica, but bound onto sepiolite to a large extent both in the presence and absence of MgCl_2_. The surface areas of Aerosil and sepiolite are not very different from that of montmorillonite K10. This indicates that surface area not sufficient to determine the adsorption characteristics of the oligonucleotides on these minerals. The weak binding on aerosil may be due to the formation of the hydrate phase with water, where strong water binding prevents biomolecules from accessing the surface H-bonding sites [36,44]. On the other hand, the binding on sepiolite was extensive, but was not dependent on the presence of MgCl_2_. Indeed, the replacement of structural Mg^2+^ inside sepiolite with different metal ions would be slow [45,46] and the predominant cations in this system would be Na^+^ and H^+^. The extensive binding may be due to the high porosity of sepiolite and/or the presence of special sites at the edges of the channels. The adsorption driving force may include hydrophobic interaction of oligonucleotide in addition to electrostatic charge interaction [47].

**Figure 4:**
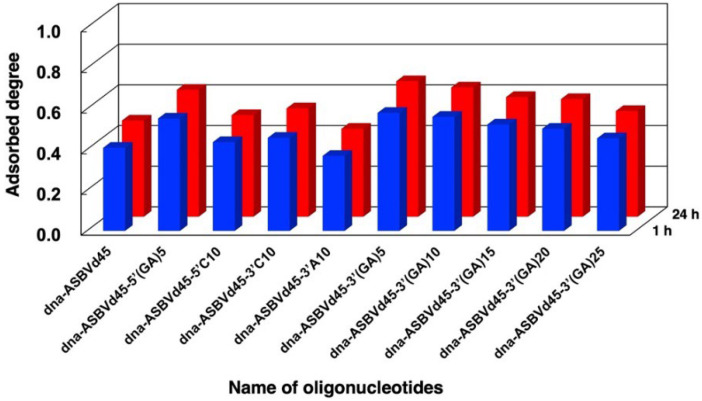
Adsorbed fractions of different model oligonucleotides (DNA) on zirconium silicate in the presence of MgCl_2_. Mineral phase/aqueous phase: 10.0 mg/2000 μL, Solutions: [DNA] = 1.0 μM, [HEPES] = 0.05 M, pH: 7.5, [MgCl_2_] = 0.05 M. Adsorption time: 1 h (blue bars), 24 h (red bars).

We examined the binding of different sequences of DNA to evaluate the role of the tail –(GA)_n_– sequence attached todna-ASBVd45 as shown in Figure 4 and Table S2 of the supplementary data. The addition of tail sequences with –C_10_– at the 3′- or 5′-terminal caused an increase in binding compared to dna-ASBVd45. The addition of repeated –(GA)_5_– sequences at the 5′-terminal induced a greater increase in binding compared to the addition of the –C_10_– sequence. In addition, the addition of –(GA)_5_– at the 3′-terminal caused a greater increase than –C_10_– or –A_10_– at the 3′-terminal. However, the additional tail sequences of –(GA)_10_– to –(GA)_25_– at 3′-terminal did not increase binding. This suggests that not all tail sequences enhance the binding of DNA molecules onto zirconium silicate. Generally, however, our findings support our assumption that tail sequences with hydrophobic bases enhance oligonucleotide binding, but the binding enhancement is dependent on whether the additional sequences are on the 3′- or 5′-terminal.

### 3.4. Binding Behaviors of Oligo(A)

**Figure 5:**
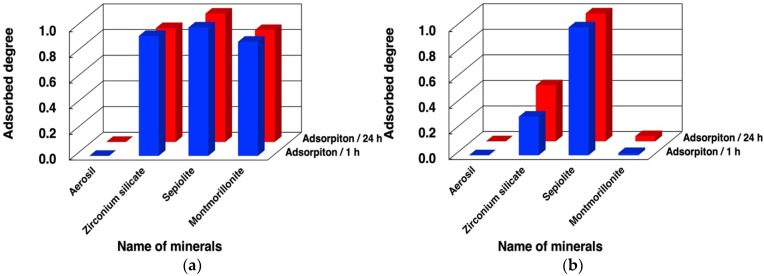
Adsorbed fraction of oligo(A) on minerals in the presence and absence of MgCl_2_. Mineral phase/aqueous phase: 20.0 mg/2000 µL, [oligoA] = 48 µM, [HEPES] = 0.05 M, pH: 7.5, [MgCl_2_]: (**a**) 0.05 M; (**b**) 0 M. Adsorption time: 1 h (blue bars), 24 h (red bars).

Using the same method, we investigated the adsorption of oligo (A) onto the four minerals. The adsorbed fraction of oligo(A) after 1 h and 24 h is summarized in Figure 5 and Table S3 of the supplementary data. The mixture of oligo(A) contained approximately 30–90-nt based on HPLC analysis. The binding behaviors were very similar to those observed for the DNA molecules tested in the present study. The presence of Mg^2+^ enhanced the binding of oligo(A) onto zirconium silicate and montmorillonite. In contrast, the binding onto Aerosil silica was much smaller both in the absence and presence of Mg^2+^, but that onto sepiolite was very high. This indicates that the binding mechanism and binding sites onto these minerals are basically the same as for the binding of DNA molecules. We believe that the binding of oligonucleotides is mainly determined by the electrostatic interaction and hydrophobic interaction between the oligonucleotides and the mineral surface, and neither of these factors was much different between the two types of polymers.

**Figure 6:**
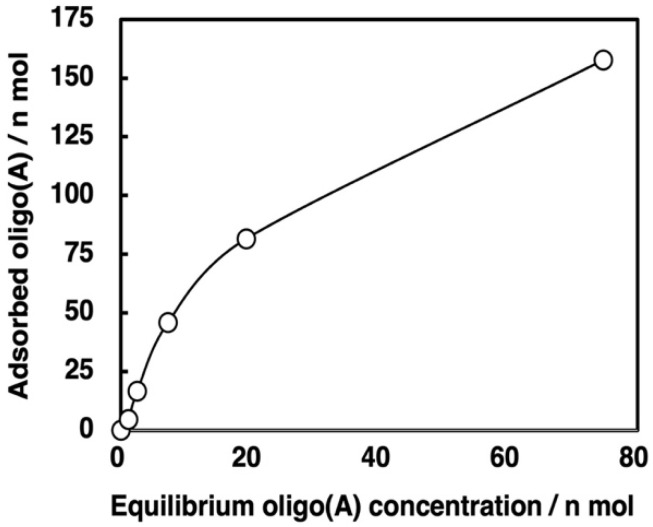
Adsorption isotherm for binding of oligo(A) on zirconium silicate. Mineral phase/aqueous phase: 10.0 mg/2000 µL, [oligoA] = 2.86–116 µM, [HEPES] = 0.05 M, pH: 7.5, [MgCl_2_] = 0.05 M, 24 h.

Based on this experimental protocol, we attempted to measure the binding isotherm of oligo(A) onto zirconium silicate; the results are shown in Figure 6 and Table S4 of the supplementary data. From the slope and intercept of the line between the plots of (mol amount of oligo(A) residue unit in supernatant)/(mol amount of oligo(A) residue unit adsorbed(vs. (mol amount of oligo(A) residue unit in supernatant) it was calculated that the saturation coverage (*a*_s_) was (2.21 ± 0.10) × 10^−7^ mol and the Langmuir adsorption coefficient (*K*_L_) was (3.29 ± 0.20) × 10^7^ M^−1^ for 10 mg zirconium silicate on the basis of the monomer unit in the oligo(A), as shown in Equation (1), where A_oligo(A),sup_ indicates the mol amount of oligo(A) residue unit in supernatant and A_oligo(A),ads_ indicates the mol amount of oligo(A) residue unit adsorbed.

A_oligo(A),sup_/A_oligo(A),ads_ = A_oligo(A),sup_/*a*_s_ + 1/(*a*_s_
*K*_L_) (1)

It is surprising that the value of *K*_L_ was fairly large. For instance, the value of *K*_L_ was greater than 10^5^ times that of the activated nucleotide monomer on montmorillonite, which might be due to the fact that rather long oligomers were used [32]. Since, to our knowledge, there are no studies of the binding of organic molecules onto zirconium silicate, this observation of the strong binding of oligonucleotides is particularly interesting. Based on the surface area of the zirconium silicate (4.82 m^2^ g^−1^) used in the present study, the exposed area for 10 mg of zirconium silicate was 482 cm^2^. The saturation coverage of 2.21 × 10^−7^ mol of adenosine residue from oligo(A) used in the experiment corresponds to 1.33 × 10^17^ adenosine residues in oligo(A) on this surface. Thus, the surface area occupied by a single adenosine residue corresponds to a square with sides of 0.60 nm, which is comparable to the size of an adenosine residue. Thus, it is estimated that the surface of zirconium silicate is covered with a monolayer of oligo(A).

The reactions using Aerosil or sepiolite had technical problems when running the self-cleavage reaction of ribozyme. In the case of Aerosil, it was difficult to separate the aqueous phase from the mineral phase. In the case of sepiolite, the desorption protocol of ribozyme from sepiolite after running the ribozyme self-cleavage reaction needs to be improved since the binding was not dependent on the presence or absence of MgCl_2_. In fact, both the procedures for stopping the reaction and desorbing the ribozyme from zirconium silicate or montmorillonite could be performed simultaneously by washing with an EDTA solution, since the ribozyme does not or partially bind on the minerals in the absence of MgCl_2_.

### 3.5. Binding Behaviors of Ribozymes and Desorption by EDTA Solution

**Figure 7:**
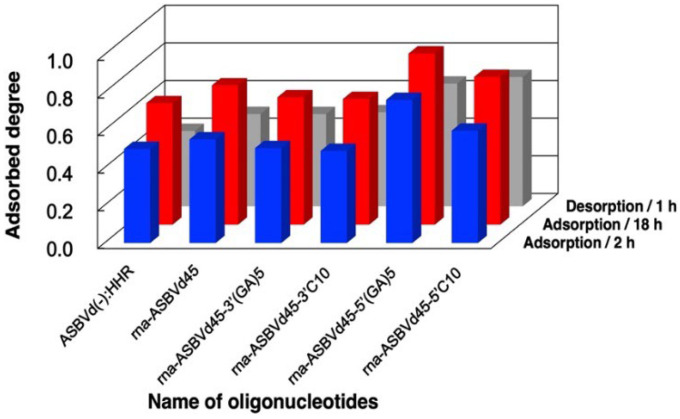
Adsorbed fractions of different model oligonucleotides (RNA) on zirconium silicate in the presence of MgCl_2_. Mineral phase/aqueous phase: 20.0 mg/2000 µL, [RNA molecules] = 20 µg/2000 µL, [HEPES] = 0.05 M, pH: 7.5, [MgCl_2_] = 0.05 M. After centrifugation, the precipitate was washed for 1 h with 1600 µL of 0.0625 M EDTA solution.

Following fundamental investigations into the binding of DNA and oligo(A), we attempted binding of the reactive ribozyme ASBVd(−):HHR and its analogues. We used six types of RNA molecules, including ASBVd(−):HHR, and examined the binding of these RNA molecules onto zirconium silicate. The binding at 2 h and 18 h is summarized in Figure 7 and Table S4 of the supplementary data. It was confirmed that ASBVd(−):HHR bound onto zirconium silicate. The adsorbed fraction increased at 18 h in contrast with DNA molecules (Figure 4) and oligo(A) (Figure 5), where the increase of binding between 1 h and 24 h was not notable. This small difference is probably due to the self-cleavage reaction of ASBVd(−):HHR. The binding of rna-ASBVd45 was somewhat greater than that of rna-ASBVd45-^3′^(GA)_5_ and rna-ASBVd45-^3′^C_10_. The binding of rna-ASBVd45-^5′^(GA)_5_ was greater than that of rna-ASBVd45 and rna-ASBVd45-^5′^C_10_. This trend was same as in the case of DNA binding with tail sequences at the 5′-terminal. In addition, this is also consistent with our speculation that the additional tail sequences, where the –(GA)_5_– sequence has a greater hydrophobicity than the –C_10_– sequence, would enhance the binding of rna-ASBVd45. On the other hand, the binding of rna-ASBVd45-^3′^(GA)_5_ and rna-ASBVd45-^3′^C_10_ was smaller than that of rna-ASBVd45. This trend is similar to that shown in the case of DNA, where these tail sequences enhance the adsorption of RNA molecules onto the zirconium silicate surface. As a general conclusion, the binding behaviors of DNA, oligo(A), and RNA molecules supports the Mg^2+^ bridge mechanism onto zirconium silicate as well as montmorillonite, where the hydrophobicity of oligonucleotides and electrostatic interaction between the charges of oligonucleotides and mineral surface are both important.

In addition, we investigated the recovery of RNA molecules upon washing with EDTA. The recovery of RNA molecules with EDTA washing is summarized in Figure 7 and Table S4 of the supplementary data. The recovery is expressed as the ratio of the recovered RNA amount to the initial RNA amount. It reached 62–88% for all six types of RNA molecules. The recovery behavior is consistent with the binding behaviors of DNA and oligo(A) in the presence and absence of MgCl_2_. As mentioned previously, washing with EDTA solution is advantageous, since EDTA enables both stopping the reaction and recovery of adsorbed RNA.

### 3.6. Self-Cleavage of ASBVd(−):HHR on Zirconium Silicate and Montmorillonite Surfaces

**Figure 8:**
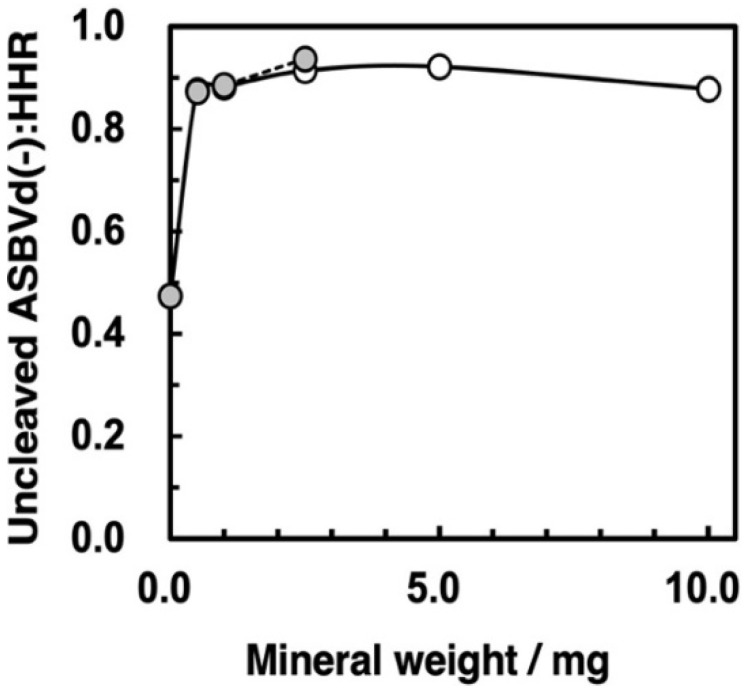
Self-cleavage degree in the presence of zirconium silicate (open circles) and montmorillonite (gray circles). Reaction conditions: Mineral phase/aqueous phase: 0–10 mg/50 µL solution, [ASBVd(−):HHR] = 3 µg/50 µL, [HEPES] = 0.05 M, pH: 7.5, [MgCl_2_] = 0.05 M, 55 °C, reaction time: 120 min. The reaction was stopped and washed by addition of 170 µL of 0.029 M EDTA solution (pH = 8.0) twice. The products were analyzed by HPLC, and the self-cleavage degree was calculated by combining the washed solutions.

Following our binding study with the ASBVd(−):HHR ribozyme, we attempted self-cleavage reactions in the presence of zirconium silicate and montmorillonite. First, we ran the self-cleavage reactions with different ratios of mineral to supernatant with a reaction time of 120 min. The ratio of remaining ASBVd(−):HHR after the 120 min reaction is plotted as a function of mineral amount in Figure 8. The pH was measured by preparing the same ratio of mineral and solution with HEPES and MgCl_2_. The addition of montmorillonite up to 2.5 mg did not change the pH of supernatant since it was controlled by HEPES buffer. For zirconium silicate, the pH did not change with up to 10 mg of mineral. The data indicate that self-cleavage was inhibited by the presence of both zirconium silicate and montmorillonite.

**Figure 9:**
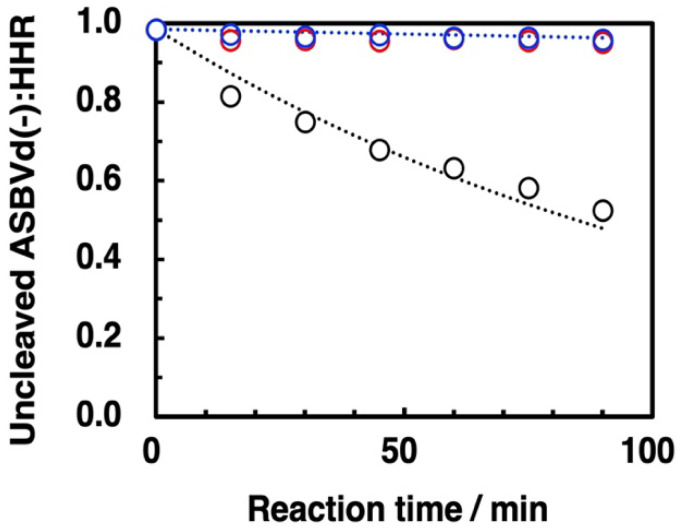
Reaction kinetic profile for the self-cleavage of ASBVd(−):HHR in the presence of zirconium silicate and montmorillonite. Reaction conditions without mineral: [ASBVd(−):HHR] = 3 µg/50 µL, [HEPES] = 0.05 M, pH: 7.5, [MgCl_2_] = 0.05 M, 55 °C, reaction time: 0–90 min. Reaction conditions with minerals: Mineral phase/aqueous phase: 2.5 mg/50 µL solution, [ASBVd(−):HHR] = 3 µg/50 µL, [HEPES] = 0.05 M, pH: 7.5, [MgCl_2_] = 0.05 M, 55 °C, reaction time: 0–90 min. The reaction was stopped and washed by addition of 170 µL of 0.029 M EDTA solution (pH = 8.0) two times. The washing solutions were analyzed by HPLC, and the self-cleavage degree was calculated by combining the washed solutions. Black open circles: control, red open circles: zirconium silicate, blue open circles: montmorillonite. Dashed lines indicate fitted lines based on the rate constants shown in Table 4.

Next, we monitored the reaction profile for reaction times of 0 to 90 min; the results are summarized in Figure 9. Here too, it is obvious that self-cleavage was inhibited strongly by both zirconium silicate and montmorillonite. The first-order rate constants of the self-cleavage reaction were calculated as shown in Table 4. The rate constant with minerals was ca. 30-fold smaller than that for the control reaction without mineral. A previous study of the influence of montmorillonite on ASBVd(−):HHR showed that the ribozyme was still active in the presence of montmorillonite although the activity was decreased by a factor of approximately 10 [15]. In the present study, the ratio of mineral to aqueous phase corresponded to 50 mg/1 mL solution, which was much higher than that in the previous study corresponding to 1 mg/mL solution, giving the different behaviors.

The amount of zirconium silicate used for the self-cleavage study was 2.5 mg, so that the adsorbed amount of nucleotide residue at saturation, based on the oligo(A) results (§ 3.4), was calculated as 5.5 × 10^−8^ mol. For comparison, the amount of ribozyme used in the present reaction was 3 µg corresponding to 9.36 × 10^−9^ mol of residues. Thus, 17% of the binding sites of zirconium silicate would be occupied with residues of the ribozyme. The total amount of the binding sites of the zirconium silicate was greater than total amount of residues of the ribozyme even if we assume that all the residues of the ribozyme bound to the surface and that the formation of three-dimensional structure of the ribozyme did not modify the occupied area. On the basis of these considerations, the apparent reaction shown in Figure 9 should reflect an almost pure reaction on the surface of zirconium silicate. For the case of montmorillonite, the surface area of montmorillonite is 2.29 × 10^2^ m^2^ g^−1^, which is 48 times greater than zirconium silicate (Table 3). So, the reaction in the presence of montmorillonite was also considered to proceed very slowly in the surface phase. This indicates that the self-cleavage reaction is strongly inhibited both on zirconium silicate and montmorillonite surfaces.

### 3.7. Role of Minerals for the Chemical Evolution of Ribozymes on Hadean Earth

According to previous studies, oligonucleotide formation by condensation is enhanced on the montmorillonite surface. Here, we concluded that the self-cleavage of ASBVd(−):HHR was inhibited on the surface of zirconium silicate (investigated for the first time) and also on the surface of montmorillonite. This would not seem to favor the idea that minerals have been active in the chemical evolution of RNA formation [15].

At the present time, the binding conformational changes of ribozyme on minerals have rarely been measured and/or assessed, although the binding of functional RNA molecules is supported by molecular conformation dynamics [48]. The inhibition of zirconium silicate for self-cleavage of the ribozyme may be due to the following. First, the conformation of the ribozyme bound on the surface zirconium silicate may not be suitable for self-cleavage. Indeed, the adsorption of biopolymers on mineral surfaces would be expected to cause a significant change in secondary structure. When adsorbing enzymes onto a surface for technological reasons, it is a challenge to conserve their secondary and tertiary structures, and complicated surface engineering strategies must be adopted for that purpose. Second, the reduction of mobility of molecules on the mineral surface would affect self-cleavage. Third, the ribozyme is adsorbed onto the surface at a fairly high density, so this may inhibit the favorable conformation of the ribozyme for self-cleavage. On the other hand, the surface area of montmorillonite is much greater than that of zirconium silicate and thus, the ASBVd(−):HHR binds on montmorillonite at a much lower density than on zirconium silicate; however, self-cleavage was also deactivated on montmorillonite. This suggests that the conditions for the self-cleavage reaction are changed on the mineral surface, presumably by a conformational change which is not favorable due to bridge binding with Mg^2+^ on mineral surfaces.

Zirconium silicate is considered to provide evidence of water on the Hadean Earth [38,39]. Thus, studying the adsorption of ribozyme and other oligonucleotides onto zirconium silicate is very interesting. It would appear that zirconium silicate is capable of concentrating oligonucleotide molecules; although the surface area is small, the adsorption coefficient is large. In addition, the inhibitory activity of the ribozyme after binding onto the zirconium silicate surface suggests that it is important to study in more depth the interaction between the mineral and functional biomolecules, as well as ribozymes. Although self-cleavage of the ribozyme is an important function for amplification of viroids in the cell, the inhibitory activity suggests that RNA molecules could have been protected from degradation on zirconium and montmorillonite surfaces. Even if self-cleavage is considered as a positive or negative feature in the evolution of primordial RNA, it is noteworthy that the activity is not reduced to zero on surfaces where the structure is not completely perturbed by adsorption.

The present study provides useful fundamental information for designing a life-like system on mineral surfaces. We showed that the enhancement of the adsorption of DNA and RNA by additional –(GA)_n_– tail sequences was partly confirmed. This is useful for designing a model RNA network system on mineral surfaces, where an alternative attachment of tail sequences is favorable for preserving biological functions of the ribozyme. In other words, this suggests the necessity for specific sequences to enhance ribozyme function on minerals.

## 4. Conclusions

We demonstrated for the first time the unexpected inhibitory activities of zirconium silicate and montmorillonite for the self-cleavage of ASBVd(−):HHR, where ASBVd(−):HHR binds onto minerals bridged with Mg^2+^ ions. This implies that the self-cleavage of ASBVd(−):HHR could be slowed by binding onto zirconium silicate and montmorillonite in the primitive deep ocean. In addition, this study showed that the binding strength measured for oligo(A), and then inferred to be similar for ASBVd(−):HHR on zirconium silicate. Since zirconium silicate is one of the oldest minerals on the ancient Earth, this study suggests that the role of minerals may have been important for the chemical evolution of RNA molecules from the very beginning of water emergence on the Hadean Earth. In addition, we successfully designed a protocol for the investigation of ribozyme reactions in the presence of minerals using a small amount of ribozyme and mineral. This will be helpful for further investigations into the relationship between the role of minerals and the chemical evolution of functional RNA molecules.

## Figures and Tables

**Table 1:** List of RNA molecules.

#	RNA Name	Sequence
1	ASBVd79(−):HHR	GGUUCUUCCC AUCUUUCCCU GAAGAGACGA AGCAAGUCGA AACUCAGAGU CGGAAAGUCG GAACAGACCU GGUUUCGUC
2	rna-ASBVd45	CUUUCCCU GAAGAGACGA AGCAAGUCGA AACUCAGAGU CGGAAAG
3	rna-ASBVd45-^3′^(GA)_5_	CUUUCCCU GAAGAGACGA AGCAAGUCGA AACUCAGAGU CGGAAAG GAGAGAGAGA
4	rna-ASBVd45-^3′^C_10_	CUUUCCCU GAAGAGACGA AGCAAGUCGA AACUCAGAGU CGGAAAG CCCCCCCCCC
5	rna-ASBVd45-^5′^(GA)_5_	GAGAGAGAGA CUUUCCCU GAAGAGACGA AGCAAGUCGA AACUCAGAGU CGGAAAG
6	rna-ASBVd45-^5′^C_10_	CCCCCCCCCC CUUUCCCU GAAGAGACGA AGCAAGUCGA AACUCAGAGU CGGAAAG

CG sequence marked in yellow is self-cleavage site of ASBVd(−):HHR or potentially self-cleavage site for RNA molecules. Single underlines indicate the core structure of the cleavage site. Double underlines indicate additional sequences for binding enhancement.

**Table 2:** List of DNA.

#	DNA Name	Sequence
1	dna-ASBVd45	CTTTCCCT GAAGAGACGA AGCAAGTCGA AACTCAGAGT CGGAAAG
2	dna-ASBVd45-^5′^(GA)_5_	GAGAGAGAGA CTTTCCCT GAAGAGACGA AGCAAGTCGA AACTCAGAGT CGGAAAG
3	dna-ASBVd45-^5′^C_10_	CCCCCCCCCC CTTTCCCT GAAGAGACGA AGCAAGTCGA AACTCAGAGT CGGAAAG
4	dna-ASBVd45-^3′^C_10_	CTTTCCCT GAAGAGACGA AGCAAGTCGA AACTCAGAGT CGGAAAG CCCCCCCCCC
5	dna-ASBVd45-^3′^A_10_	CTTTCCCT GAAGAGACGA AGCAAGTCGA AACTCAGAGT CGGAAAG AAAAAAAAAA
6	dna-ASBVd45-^3′^(GA)_5_	CTTTCCCT GAAGAGACGA AGCAAGTCGA AACTCAGAGT CGGAAAG GAGAGAGAGA
7	dna-ASBVd45-^3′^(GA)_10_	CTTTCCCT GAAGAGACGA AGCAAGTCGA AACTCAGAGT CGGAAAG GAGAGAGAGA GAGAGAGAGA
8	dna-ASBVd45-^3′^(GA)_15_	CTTTCCCT GAAGAGACGA AGCAAGTCGA AACTCAGAGT CGGAAAG GAGAGAGAGA GAGAGAGAGA GAGAGAGAGA
9	dna-ASBVd45-^3′^(GA)_20_	CTTTCCCT GAAGAGACGA AGCAAGTCGA AACTCAGAGT CGGAAAG GAGAGAGAGA GAGAGAGAGA GAGAGAGAGA GAGAGAGAGA
10	dna-ASBVd45-^3′^(GA)_25_	CTTTCCCT GAAGAGACGA AGCAAGTCGA AACTCAGAGT CGGAAAG GAGAGAGAGA GAGAGAGAGA GAGAGAGAGA GAGAGAGAGA GAGAGAGAGA

CG sequence marked in yellow is the correspondent site for the self-cleavage of the ASBVd(−):HHR. Double underlines indicated additional sequences for binding enhancement.

**Table 3:** BET surface area of minerals.

Minerals	Area (BET)/m^2^ g^−1^
Aerosil	3.69 × 10^2^
Sepiolite	3.51× 10^2^
Zirconium silicate	4.82 × 10^0^
Montmorillonite	2.29 × 10^2^

**Table 4:** First-order rate constants for self-cleavage of ASBVd(−):HHR.

Conditions	*k*/min^−1^ *
Control	(80.0 ± 11.0) × 10^−4^
In the presence of zirconium silicate	(2.46 ± 1.12) × 10^−4^
In the presence of montmorillonite	(2.38 ± 0.55) × 10^−4^

* Error ranges are the standard deviation derived from straight line of the first-order kinetic plots for the reaction profiles shown in Figure 9.

## Data Availability

Not applicable.

## Supplementary Materials

The following supporting information can be downloaded at: https://www.mdpi.com/article/10.3390/life12111689/s1. Table S1a: Adsorption degree of dna-ASBVd45 on minerals in the presence of MgCl_2_. Table S1b: Adsorption degree of dna-ASBVd45 on minerals in the absence of MgCl_2_. Table S2: Binding degree of different DNA molecules on zirconium silicate; Table S3a: Adsorption degree of oligo(A) on minerals in the presence of MgCl_2_. Table S3b: Adsorption degree of oligo(A) on minerals in the absence of MgCl_2_. Table S4: Binding degree of oligoA on zirconium silicate. Table S5: Binding degree and recovery degree of RNA molecules on zirconium silicate.
