# Supplementary material for: Life on Minerals: Binding Behaviors of Oligonucleotides on Zirconium Silicate and Its Inhibitory Activity for the Self-Cleavage of Hammerhead Ribozyme
Source: Life (Basel). 2022 Oct 24;12(11):1689. doi: 10.3390/life12111689 (PMC9697215; doi:10.3390/life12111689)
Supplement: Supplementary file 1 [file life-12-01689-s001.zip › life-1972463-supplementary.pdf]

Supplementary data

Table S1a. Adsorbed fraction of dna-ASBVd45 on minerals in the presence of $MgCl_2$.

	1 h	24 h
Aerosil	0.015	0.010
Zirconium silicate	0.545	0.596
Sepiolite	0.985	0.987
Montmorillonite	0.906	0.941

Mineral phase / Aqueous phase: 20.0 mg / 2000 μL, [dna-ASBVd45] = 1.14 μM, [HEPES] = 0.05 M, pH: 7.5, [$MgCl_2$] = 0.05 M.

Table S1b. Adsorbed fraction of dna-ASBVd45 on minerals in the absence of $MgCl_2$.

	1 h	24 h
Aerosil	No binding	No binding
Zirconium silicate	0.111	0.137
Sepiolite	0.966	0.993
Montmorillonite	No binding	0.018

Mineral phase / Aqueous phase: 20.0 mg / 2000 μL, [dna-ASBVd45] = 1.14 μM, [HEPES] = 0.05 M, pH: 7.5.

Table S2. Adsorbed fraction of different DNA molecules on zirconium silicate.

DNA	1 h	24 h
dna-ASBVd45	0.409	0.473
dna-ASBVd45-$^{5'}(GA)_5$	0.553	0.625
dna-ASBVd45-$^{5'}C_{10}$	0.437	0.500
dna-ASBVd45-$^{3'}C_{10}$	0.458	0.533
dna-ASBVd45-$^{3'}A_{10}$	0.369	0.433
dna-ASBVd45-$^{3'}(GA)_5$	0.581	0.667
dna-ASBVd45-$^{3'}(GA)_{10}$	0.561	0.636
dna-ASBVd45-$^{3'}(GA)_{15}$	0.524	0.589
dna-ASBVd45-$^{3'}(GA)_{20}$	0.503	0.579
dna-ASBVd45-$^{3'}(GA)_{25}$	0.455	0.520

Mineral phase / Aqueous phase: 10.0 mg / 2000 μL, Solutions : [DNA] = 1.0 μM, [HEPES] = 0.05 M, pH: 7.5, [$MgCl_2$] = 0.05 M.

Table S3a. Adsorbed fraction of oligo(A) on minerals in the presence of $MgCl_2$.

	1 h	24 h
Aerosil	No binding	No binding
Zirconium silicate	0.934	0.887
Sepiolite	1.005	1.005
Montmorillonite	0.889	0.872

Mineral phase / Aqueous phase: 20.0 mg / 2000 μL, [oligoA] = 48 μM, [HEPES] = 0.05 M, pH: 7.5, [$MgCl_2$] = 0.05 M.

Table S3b. Adsorption degree of oligo(A) on minerals in the absence of $MgCl_2$.

	1 h	24 h
Aerosil	No binding	No binding
Zirconium silicate	0.302	0.438
Sepiolite	1.020	1.018
Montmorillonite	0.017	0.041

Mineral phase / Aqueous phase: 20.0 mg / 2000 μL, [oligoA] = 48 μM, [HEPES] = 0.05 M, pH: 7.5.

Table S4. Binding degree of oligoA on zirconium silicate.

#	Initial Conc. of oligoA (M)	mol supernatant	mol adsorbed
1	2.86×10^{-6}	1.14×10^{-9}	4.57×10^{-9}
2	9.59×10^{-6}	2.51×10^{-9}	1.67×10^{-8}
3	2.66×10^{-5}	7.31×10^{-9}	4.59×10^{-8}
4	5.05×10^{-5}	1.94×10^{-8}	8.17×10^{-8}
5	1.16×10^{-4}	7.47×10^{-8}	1.58×10^{-7}

Mineral phase / Aqueous phase: 10.0 mg / 2000 μL, [oligoA] = 2.86 – 116 μM, [HEPES] = 0.05 M, pH: 7.5, [$MgCl_2$] = 0.05 M, 24 h.

Table S5. Binding degree and recovery degree of RNA molecules on zirconium silicate.

RNA	Binding at 2 h	Binding at 18 h	Recovery by EDTA washing
ASBVd79(-):HHR	0.500	0.645	0.400
rna-ASBVd45	0.551	0.740	0.490
rna-ASBVd45-$^{3'}$(GA)$_5$	0.504	0.677	0.490
rna-ASBVd45-$^{3'}$C$_{10}$	0.489	0.668	0.500
rna-ASBVd45-$^{5'}$(GA)$_5$	0.761	0.909	0.652
rna-ASBVd45-$^{5'}$C$_{10}$	0.597	0.785	0.687

Mineral phase / Aqueous phase: 20.0 mg / 2000 μL, [RNA molecules] = 20 μg / 2000 μL, [HEPES] = 0.05 M, pH: 7.5, [$MgCl_2$] = 0.05 M. Washed for 1 h with 1600 μL of 0.0625 M EDTA solution.
